# Associations of Changes in Alcohol Consumption on the Risk of Depression/Suicide Among Initial Nondrinkers

**DOI:** 10.1155/2024/7560390

**Published:** 2024-11-11

**Authors:** Sungmin Cho, Sangwoo Park, Su Kyoung Lee, Si Nae Oh, Kyae Hyung Kim, Ahryoung Ko, Sang Min Park

**Affiliations:** ^1^Department of Biomedical Sciences, Seoul National University Graduate School, Seoul, Republic of Korea; ^2^Institute of Health Informatics, University College London, London, UK; ^3^Institute of Health and Environment, Graduate School of Public Health, Seoul National University, Seoul, Republic of Korea; ^4^Department of Family Medicine, National Health Insurance Ilsan Hospital, Goyang, Republic of Korea; ^5^Home Healthcare Clinic, Public Healthcare Center, Department of Family Medicine, Seoul National University Hospital, Seoul, Republic of Korea; ^6^Department of Public Healthcare, Seoul National University Hospital, Seoul, Republic of Korea; ^7^Department of Family Medicine, Seoul National University Hospital, Seoul, Republic of Korea

## Abstract

Although prior studies showed the association between the amount of alcohol ingestion and the risk of depression and suicide, there has been a lack of research considering changes in alcohol intake over time. This research aimed to assess the associations of alcohol consumption level changes and the risk of depression and suicide among initial nondrinkers. Using data from the National Health Insurance Service in South Korea between 2002 and 2019, a total of 129,446 subjects were included and monitored from January 1, 2011, to December 31, 2019, of which 102,721 were never drinkers and 26,725 were former drinkers. For depression, the follow-up periods ranged from 0.01 to 9.00 years (mean 8.65, median 9.00 years). Moreover, for suicide, the follow-up periods ranged from 0.03 to 9.00 years (mean 8.99, median 9.00 years). To ensure robust results, the model was adjusted for several confounders in three steps: Model 1 was adjusted for sociodemographic factors (age and sex), Model 2 included additional lifestyle factors (household income, smoking status, and physical activity) in addition to Model 1 variables, and Model 3 included all variables from Model 2 and incorporated further variables including body mass index (BMI), systolic blood pressure, fasting serum glucose, total cholesterol, and the Charlson Comorbidity Index (CCI). The Cox proportional hazard regression was utilized to estimate the adjusted hazard ratio (aHR) and 95% confidence intervals (CIs) for depression and suicide risk after an increase in alcohol consumption. Individuals who increased alcohol consumption lightly up to one glass per day had a reduced risk of depression (aHR, 0.91; 95% CI, 0.84–0.98) compared with individuals who maintained their nondrinking status at the third medical checkup. Notably, stratified analyses indicated that the associations were only evident in those younger than 60 years and those physically active. Additionally, among former drinkers, those who increased their alcohol intake to four or more glasses per day had an increased risk of depression (aHR, 1.31; 95% CI, 1.04–1.66). However, individuals who initiated drinking between two and four glasses of alcoholic beverages per day were found to have a higher risk of suicide (aHR, 2.25; 95% CI, 1.31–3.87) relative to those who continued to abstain from drinking. Our findings suggest that small increases in alcohol intake among the initial nondrinkers are associated with a reduced risk of depression, whereas moderate-to-heavy increments in alcohol consumption are associated with a detrimental risk of suicide. This study has several limitations including the low number of suicide events, reliance on self-reported alcohol consumption which may introduce underreporting bias, and the exclusion of important confounding variables such as educational attainment and dietary factors. Furthermore, the study population was exclusively Korean, limiting the generalizability of the findings to other ethnic groups.

## 1. Introduction

Alcohol is a widely used toxic and psychoactive intoxicant which may foster dependence or addiction in the world [1]. Alcohol consumption has been perceived as a notable risk factor for avoidable health issues and mortality, comprising 12.2% and 3.8% of the deaths in males and females, respectively, among the global population aged from 15 to 49 in 2016 [2]. Despite its inherent risks, it has garnered social acceptance in numerous cultures for centuries due to its properties of relaxation and pleasure [1]. In 2016, South Korea had a notable alcohol consumption rate, with an average of 10.2 L per person per year, which was the second highest among Asian countries [3]. In addition, the National Collaborating Centre for Mental Health [1] highlighted the connection between alcohol consumption and various mental and behavioral disorders. Depression has been on the rise globally, with incidents soaring from 172 million in 1990 to 258 million in 2017, marking a 49.9% increment [4]. Furthermore, suicide is a substantial public health challenge worldwide, with over 800,000 people dying by suicide each year [5]. In 2011, South Korea's statistics showed a suicide rate of 31.7 per 100,000 people, positioning it as the country's fourth highest cause of mortality [6]. A number of research have firmly established the association between alcohol intake and its consequent impact on depression and suicide [7–11]. However, these studies on the relationship between alcohol intake and mental health have only examined alcohol intake only at baseline. To date, there has been no investigation considering changes in drinking status over the study period and its association with depression and suicide. This study is motivated by the crucial necessity to comprehend how increases in alcohol consumption by nondrinkers over time may influence mental health outcomes, particularly in a high-risk population where cultural practices and social norms around alcohol are deeply entrenched.

## 2. Materials and Methods

### 2.1. Data Source

The National Health Insurance Service (NHIS) in the Republic of Korea (hereafter “Korea”), a nonprofit organization under the administration of the Korean government, offers health insurance coverage to Korean citizens, yielding an enrollment rate of 98% [12]. The National Health Insurance Service-Health Screening Cohort (NHIS-HEALS) comprises individuals aged 40 or above who participate in the biennial general national health examination program of the NHIS. This research utilized the data sourced from the NHIS-HEALS between 2002 and 2019 for the statistical analysis. The NHIS-HEALS dataset (NHIS-2023-2-194) retains various medical information, including sociodemographic factors, hospital utilization, results of medical examinations, and pharmaceutical prescriptions. Given the NHIS's provision of its database for the purpose of research, numerous epidemiological studies have been conducted, and its credibility and validity have been substantiated and expounded in other sources [13].

### 2.2. Study Population and Design

**Figure 1:**
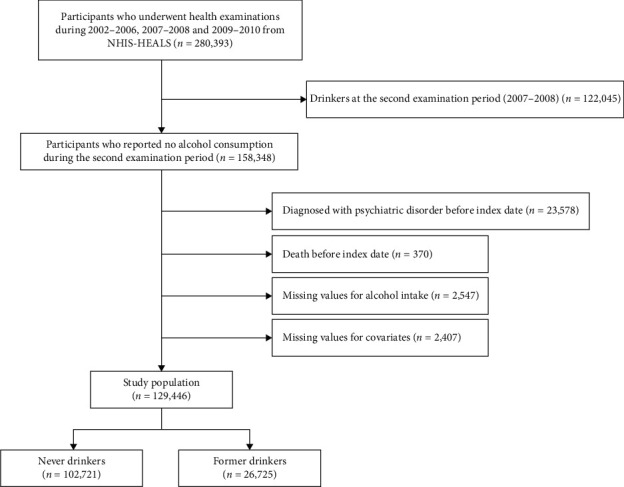
Title flow diagram of the study subjects. NHIS-HEALS, National Health Insurance Service-Health Screening Cohort.

The study cohort consisted of Korean adults aged 40 years or above who underwent health examinations during the periods 2002–2006, 2007−2008, and 2009–2010. Participants were included if they participated in all three health screening periods. From the initial pool of 280,393 participants, individuals were excluded if they reported alcohol consumption during the second examination period (*n* = 122,045). Further exclusions were made for individuals with a documented psychiatric diagnosis (*n* = 23,578) or those whose death was recorded before the index date (January 1, 2011) (*n* = 370). Additionally, participants with missing data regarding alcohol consumption habits (*n* = 2547) or pertinent covariate variables (*n* = 2407) were also excluded. Consequently, a final cohort of 129,446 participants was considered for analysis. The selection process for the study cohort is outlined in Figure 1.

Within this cohort, participants were classified as never drinkers if they reported no alcohol consumption during the 2002–2006 baseline period (*n* = 102,721). Those who reported any alcohol consumption during this period were classified as former drinkers (*n* = 26,725). These individuals were prospectively monitored from the index date of January 1, 2011, until the occurrence of depression/suicide and mortality or until December 31, 2019, whichever occurred earlier.

### 2.3. Key Variables

In this study, the evaluation of the volume of alcohol consumption was undertaken employing two methodologies. The primary method was to categorize it into different levels by the number of glasses per day: nondrinking (0), >0–≤1, >1–≤2, >2–≤4, and >4. This categorization of alcohol consumption aligns with the methodology used by previous study [14]. Regardless of beverage type, a standard single-serving unit was standardized to contain 10 g of ethanol [15]. Additionally, the secondary method was to categorize the alcohol ingestion based on the frequency of weekly alcohol consumption: 0, 1, 2, 3–4, and 5−7 days per week. According to Abel, Kruger, and Friedl [16], light drinking corresponds to the consumption of 1.2 drinks per day, moderate drinking refers to consuming 2.2 drinks per day, and heavy drinking is defined as 3.5 drinks or more per day. Therefore, in this study, the amount of alcohol consumption is split into five categories: nondrinking, light drinking (>0–≤1 glass per day and 1 day per week), light-to-moderate drinking (>1–≤2 glasses and 2 days), moderate-to-heavy drinking (>2–≤4 glasses and 3–4 days), and heavy drinking (>4 glasses and 5–7 days). The first outcome in this investigation encompassed the incident of depression over the period of follow-up, defined by the International Classification of Diseases, 10th version (ICD-10) codes of F32 and F33 signifying major depressive disorder with a single and recurrent episode, along with the records of antidepressant prescription. Analogously, the secondary outcome, suicide, was defined by the death with diagnostic codes (ICD-10 codes: intentional self-harm, X60–X84, and sequelae of intentional self-harm, Y87.0). The adoption of ICD-10 codes for depression and suicide was consistent with a precedent study [17, 18].

### 2.4. Covariates

The analysis included several covariates sourced from the NHIS-HEALS dataset to control for potential confounders: sex, age, household income, smoking status, physical activity, body mass index (BMI), systolic blood pressure, triglyceride levels, fasting serum glucose levels, total cholesterol, and the Charlson Comorbidity Index (CCI) [13]. The widely recognized CCI was used as a risk adjustment measure for multiple comorbidities by using each individual's disease diagnosis records (ICD-10 codes) to calculate the CCI [14].

Household income was categorized according to the income quantile system established by the South Korean government, which divides the population into 10 groups based on household income. This system, updated annually by the Ministry of Health and Welfare, is calculated based on the median income, which varies by the number of household members. For this study, these quantiles were further consolidated into four groups: the highest income group (ninth and tenth groups) was classified as the first group, the sixth to eighth groups as the second group, the third to fifth groups as the third group, and the lowest income group (first and second groups) along with recipients of medical aid were classified as the fourth group.

Physical activity was calculated by averaging responses to two questions: the number of days per week of vigorous exercise lasting at least 20 min and the number of days per week of moderate exercise lasting at least 30 min. Furthermore, smoking status was assessed through a questionnaire and categorized into three groups: (1) never smoker, (2) former smoker, and (3) current smoker. Systolic blood pressure was measured in mmHg, and BMI was calculated using weight and height, all collected during the health examination. In addition, blood samples were collected following an overnight fast, and fasting serum glucose, triglyceride, and total cholesterol levels were measured in mg/dL [19].

### 2.5. Statistical Analysis

Cox proportional hazard regression models were employed to calculate adjusted hazard ratios (aHRs) along with their corresponding 95% confidence intervals (CIs) for incidence of depression and suicide, considering changes in the volume of alcohol consumption measured in terms of both daily glasses and weekly frequency. Subsequently, stratified analyses were conducted to scrutinize the association between changes in alcohol ingestion and the risk of experiencing depression and suicide. The stratification was based on sex, age, physical activity, income level, smoking status, and the CCI. Additionally, these stratified analyses were conducted by categorizing each variable separately to calculate aHRs. However, the *p*-values for interaction were derived using a regression model with multiplicative interaction terms.

All statistical analyses, as well as data collection and processing, were executed using SAS version 9.4 (SAS Institute, Cary, NC, USA). A significance level of ≤0.05 (two-sided) was employed to establish statistical significance.

### 2.6. Institutional Review Board (IRB) Approval

The IRB of Seoul National University Hospital granted approval for this study (IRB number: E-2305-050-1429). Due to the anonymized NHIS-HEALS database with strict confidentiality guidelines before distribution, the requirement for informed consent was waived.

## 3. Results

### 3.1. Baseline Characteristics

Among the initial pool of 129,446 nondrinkers, the majority (85.3%) of participants remained abstinent during the third health examination period. However, 12,087 (9.3%) initiated drinking alcohol lightly (>0–≤1 glass per day), 3026 (2.3%) adopted light-to-moderate consumption (>1–≤2 glasses per day), 2363 (1.8%) engaged in moderate-to-heavy alcohol intake (>2–≤4 glasses per day), and 1523 (1.2%) exceeded four glasses per day.  Table 1 provides a detailed overview of the baseline characteristics of the total population based on daily alcohol consumption. Table S1 presents these characteristics separately for never drinkers and former drinkers. Additionally, Table S2 provides a descriptive analysis of the study population based on the number of days of alcohol consumption per week, offering another perspective on the drinking patterns.

The mean age of participants was 59.7 (±9.0) years, with females constituting 61.8% of the cohort. Notably, 90.3% reported a nonsmoking status, while 51.2% indicated no regular physical activity. Furthermore, individuals who increased alcohol ingestion were found to have a higher possibility of being male, have a history of smoking or currently smoke, and engage in physical activity compared to those who retained the abstinent drinking status. Additionally, there was an observable trend toward increased BMI, systolic blood pressure, triglyceride, and fasting serum glucose levels with increased alcohol consumption, while total cholesterol levels tended to decrease.

Among the total study population, never drinkers (79.4%) were nearly four times more prevalent than former drinkers (20.6%). Never drinkers demonstrated a higher percentage of never smokers (83.2%) and females (69.4%). In contrast, former drinkers had a higher possibility of past or current smoking, with 18.9% being current smokers, and a greater percentage of males (67.4%). Additionally, former drinkers also exhibited higher physical activity levels compared to never drinkers.

### 3.2. Association of Changes in Alcohol Consumption and Depression/Suicide

The results of the relationship between increments in alcohol ingestion, categorized by daily drink count, and the risk of depression and suicide are presented in Table 2. During the follow-up period, among those who sustained their nondrinking status in the third health assessment, a total of 8537 individuals experienced depression over 954,129 person-years, and 182 subjects died by suicide over 992,788 person-years.

Individuals who increased alcohol ingestion to light drinking showed a statistically significant decreased risk of depression compared to persistent nondrinkers (aHR, 0.91; 95% CI, 0.84–0.98). Additionally, those who increased to light-to-moderate drinking showed a null association with a reduced risk (aHR, 0.94; 95% CI, 0.80–1.10). However, individuals increasing their alcohol intake to moderate-to-heavy drinking did not show a significant impact on depression risk overall, although there was a nonsignificant trend toward increased risk among never drinkers (aHR, 1.28; 95% CI, 0.90–1.81). Moreover, increasing to heavy drinking was associated with an increased risk of depression, with a statistically significant result observed particularly in former drinkers (aHR, 1.31; 95% CI, 1.04–1.66), suggesting a notable concern for this group.

For the risk of suicide, individuals who increased their alcohol intake to light drinking exhibited a null association with overall risk (aHR, 0.98; 95% CI, 0.64–1.50). Furthermore, those who increased to light-to-moderate drinking showed a null association with a slight increase in risk of suicide (aHR, 1.11; 95% CI, 0.56–2.18). Importantly, individuals who increased their intake to moderate-to-heavy drinking were significantly associated with more than a twofold increase in suicide risk (aHR, 2.25; 95% CI, 1.31–3.87).

In addition, Table 3 indicates the hazard ratios for the risk of depression and suicide based on changes in alcohol ingestion frequency per week. In alignment with the results presented in Table 2, compared to individuals who continued their zero-alcohol consumption status, elevating alcohol consumption to 1 day per week was associated with a reduced risk of depressive disorders (aHR, 0.88; 95% CI, 0.80–0.96). However, an approximately doubled risk of suicide was observed in the individuals who changed alcohol consumption from abstaining to drinking 3–4 days per week (aHR, 2.03; 95% CI, 1.20–3.43). These consistent patterns across both tables strengthen the validity of our findings, demonstrating a clear association between increased alcohol consumption and the risks of depression and suicide.

Moreover, increasing alcohol consumption to 2 days per week showed a null association with reduced risk of depression (aHR, 0.96; 95% CI, 0.83–1.10), as this result was not statistically significant. Similarly, increasing alcohol consumption to 5–7 days per week showed a null association with increased risk of suicide (aHR, 1.57; 95% CI, 0.82–3.00), which nonetheless highlights a potential area of concern.

Table 4 presents the aHRs for depression and suicide associated with increments of one drink per day and 1 day per week in alcohol intake. The results indicate that, for depression, increasing alcohol intake by one drink per day or one additional drinking day per week does not significantly alter the risk. However, for suicide, the data show that each additional drink per day or each additional drinking day per week increases the risk of suicide by approximately 1.2 times (aHR, 1.17; 95% CI, 1.02–1.34; aHR, 1.15; 95% CI, 1.02–1.30).

### 3.3. Stratified Analysis on the Association of Increases in Alcohol Intake and the Risk of Depression

The findings of the stratified analysis examining the association between increases in alcohol consumption, categorized by both daily glass intake and weekly frequency, and the risk of depression are outlined in Table 5 and Tables S3 and S4. These analyses were performed within subgroups defined by sex, age, physical activity, household income, smoking habits, and medical comorbidities.

In Table 5, significant interactions were observed in the age and physical activity subgroups. For the age subgroup, participants under 60 years showed a significantly reduced risk of depression with a light increase in alcohol consumption (aHR, 0.85; 95% CI, 0.76–0.95) and a *p* for interaction value of 0.02. This indicates a reduced risk of depression in this age group with low levels of alcohol intake increment. Conversely, for those aged 60 years and older, the association was not statistically significant.

For physical activity, individuals engaging in regular physical activity also demonstrated a significant interaction. Those in the physically active subgroup showed a reduced risk of depression (aHR, 0.87; 95% CI, 0.78–0.97) with a *p* for interaction value of 0.03.

Additionally, in the smoking status subgroup, current non-smokers who increased alcohol consumption to >4 drinks per day had a significantly increased risk of depression (aHR, 1.33; 95% CI, 1.04–1.70), indicating a detrimental association of high alcohol intake in this group.

These findings underscore the importance of considering individual characteristics such as age and physical activity when assessing the relationship between alcohol consumption and depression risk.

## 4. Discussion

In this research, we investigated how increases in alcohol consumption influenced the risk of depression and suicide in a cohort of initially nondrinking Korean adults. The findings of this study indicate that the increase in drinking patterns from nondrinking to light drinking (>0–≤1 glass per day and 1 day per week) is associated with a reduced risk of depression. However, increments to moderate-to-heavy alcohol consumption (>2–≤4 glasses per day and 3 or 4 days per week) have a possibility to lead to suicide. We used a reference group of participants who continued nonalcohol drinking status in the third medical examination. The results of this study could inform strategies to mitigate and manage depression and suicide attempts, particularly relevant to societies such as Korea, where alcohol use is notably high [3]. In addition, a slight increase in alcohol intake could potentially have some positive impacts on mental health, but due to the potential harms of alcohol misuse, which can lead to suicide, initiating alcohol consumption cannot be recommended as a preventive measure for depression among nondrinkers. Instead, light drinking for social interaction only can be recommended.

Prior studies have examined the relationship between alcohol consumption and depressive symptoms, demonstrating a nonlinear association in which individuals classified as light or moderate drinkers had a lower possibility of being depressed in comparison to nondrinkers and heavy drinkers [7, 8, 20, 21]. The statistical analysis of this study confirms the findings of previous research regarding the nonlinear relationship between the consumption of alcohol and depression. Moreover, other previous research demonstrated that excessive alcohol consumption can lead to suicidal behavior [10, 11]. However, in these studies, alcohol ingestion was measured only once at baseline and did not account for changes in drinking status over time.

A potential rationale that could clarify the association between light alcohol intake and a lower risk of depression is that social drinking may reflect or facilitate healthy social interaction and bonding, which is a well-known protective factor for depression [22–24]. Social engagement and positive interpersonal relationships may buffer against stress, enhance psychological well-being, and even reduce depressive symptoms [25, 26]. In addition, another possible biological mechanism is the modulation of brain-derived neurotrophic factor (BDNF), which is linked to the development of depression [22, 27]. Moderate alcohol ingestion has been shown to increase the expression and secretion of BDNF and decrease inflammatory biomarkers, which may have neuroprotective and anti-inflammatory effects on the brain [22, 28]. While the mechanisms previously described pertain to moderate drinking, we postulate that the same underlying mechanisms are applicable to light drinking in the context of our study, reflecting a consistent association with reduced risk of depression.

However, heavy drinking leading to intoxication has been shown to increase the levels of aggression, which plays a significant role in suicidal tendencies [29]. Multiple evidence indicates an association between alcohol consumption, depletion of serotonin (5-hydroxytryptamine) levels, and aggression [30–32]. 5-Hydroxyindoleacetic acid (5-HIAA) is the primary serotonergic metabolite in the central nervous system, and its levels have been employed as an indicator of serotonergic activity [33, 34]. Previous research [35, 36] found that acute and chronic alcohol exposure can lead to a reduction in serotonin levels (5-HIAA) in the brain. In addition, there is an association between reduced serotonin and aggression [32]. Therefore, reduced serotonin activity would increase aggression, ultimately enhancing the risk of engaging in suicidal behavior [29, 33, 34].

It is noteworthy to mention that we observed in our stratified analysis individuals who identified as nonsmokers at the second medical checkup exhibited an elevated risk of depression when their alcohol consumption surpassed moderate levels, as clearly demonstrated in Table 5. One possible explanation of this phenomenon is that depression has significantly increased from 2005 to 2013 among all current, former, and never smokers [37]. However, the increase among former and never smokers was even more prominent compared to current smokers [37]. In addition, Hughes [38] stated that quitting smoking appears to be associated with major depression in some individuals, potentially leading to suicidal tendencies. However, it is crucial to note that no prior studies have investigated the associations of increased alcohol consumption among former and never smokers on their mental health. Consequently, there is a critical need for further research in this area.

### 4.1. Study Strengths and Limitations

This research has several notable strengths. Firstly, extensive covariate adjustments, including demographic factors, socioeconomic status, health-related behavior, laboratory findings, and medical comorbidities, were accounted for in the multivariable analyses. Furthermore, unlike prior studies [21] that relied on self-reported measures such as Patient Health Questionnaire-9 (PHQ-9), our research successfully examined the association between the changes in alcohol consumption and depression risk by employing verified clinical diagnoses in the general population. Moreover, in previous studies, alcohol ingestion was measured only once at baseline and did not account for changes in drinking status over time. The present study addressed this gap by investigating the associations between changes in alcohol intake and the risk of depression and suicide within a cohort of Korean adults who underwent health checkups at three time points.

Nevertheless, it is crucial to acknowledge that this study has various limitations. Firstly, distinguishing never drinkers and former drinkers was challenging, as former drinkers were identified based on any alcohol consumption reported during the initial health examination period (2002–2006), while never drinkers reported no drinking during this period. In addition, due to data limitations, we were unable to include variables such as educational attainment, dietary factors (e.g., energy intake and consumption of key foods or nutrients), and neuropsychiatric symptoms (e.g., baseline depressive symptoms), which could potentially confound the relationship between alcohol consumption and mental health outcomes.

This study relied on information about self-reported alcohol consumption, which may be subject to underreporting and recall bias [39]. Although self-reported assessments are generally reliable for light-to-moderate drinkers, heavy drinkers tend to underestimate their consumption [40]. Given that only approximately 1% of participants reported consuming over four glasses of alcohol daily or drinking 5 or more days per week, any potential underreporting likely had minimal influence on our findings. Episodic binge drinking could also impact the study's outcomes, but the limited number of participants consuming over four glasses daily precluded meaningful comparisons between every day and occasional drinkers.

Moreover, we could not exclude former drinkers who had ceased alcohol consumption due to comorbidities, despite the study cohort initially excluding individuals with pre-existing psychiatric disorders. Additionally, we were unable to consider changes in covariates over time, such as employment status, income, or other significant life events, which may influence the association between alcohol intake and mental health. Furthermore, we acknowledge that increased alcohol consumption may be associated with the risk of developing other conditions such as liver disease, heart disease, and cancer, which may be associated with depression or suicide incidence. However, the analysis was constrained to adjust for social factors and CCI as covariates only at the time of the third health screening period.

A notable constraint of this study is the scarce suicide events. Due to this limitation, we did not analyze the associations with suicide in never and former drinkers separately. Consequently, stratified analyses were conducted only for depression. This constraint emphasizes the necessity for future research employing larger sample sizes or extended follow-up periods in order to gain a more comprehensive understanding of the association between changes in alcohol intake and the risk of suicide. Finally, the study population was exclusively Korean, potentially limiting the applicability of the results to other countries and ethnic groups. Therefore, further research is needed to investigate how changes in drinking habits are associated with the incidence of depression and suicide within populations of diverse ethnic backgrounds.

## 5. Conclusions

In conclusion, this study examined the association of changes in alcohol intake and the risk of depression and suicide in a cohort of Korean adults and found that light drinking was associated to a lower risk of depression, whereas moderate-to-heavy drinking was linked to a higher risk of suicide. However, it is important to note that this finding might be confounded by selection bias and the presence of individuals who quit drinking due to health issues in the reference group. The study also had several limitations, such as the use of self-reported data, the lack of information on drinking patterns, and the limited generalizability to other populations and ethnicities. Therefore, more rigorous and comprehensive future studies are needed to confirm the causal relationship between increases in drinking status and depression and suicide, as well as to explore the potential mechanisms and moderators of this association.

## Figures and Tables

**Table 1:** Descriptive statistics of the participants in the National Health Insurance Service (alcohol consumption categorized by the number of glasses per day).

Participant characteristics	Alcohol intake during the second health examination (drinks per day)					
	Total	0	>0–≤1	>1–≤ 2	>2–≤4	>4
Number of participants (%)	129,446	110,447 (85.3)	12,087 (9.3)	3026 (2.3)	2363 (1.8)	1523 (1.2)
Age, years, mean (SD)	59.7 (9.0)	60.1 (9.0)	57.8 (8.4)	57.7 (8.3)	57.9 (8.3)	58.1 (8.5)
Sex, *N* (%)						
Men	49,474 (38.2)	35,505 (32.2)	7686 (63.6)	2644 (87.4)	2180 (92.3)	1459 (95.8)
Women	79,972 (61.8)	74,942 (67.9)	4401 (36.4)	382 (12.6)	183 (7.7)	64 (4.2)
Household income^a^, *N* (%)						
First (highest)	44,158 (34.1)	36,951 (33.5)	4602 (38.1)	1187 (39.2)	890 (37.7)	528 (34.7)
Second	37,837 (29.2)	32,230 (29.2)	3451 (28.6)	918 (30.3)	730 (30.9)	508 (33.4)
Third	27,638 (21.4)	23,919 (21.7)	2399 (19.9)	575 (19.0)	439 (18.6)	306 (20.1)
Fourth (lowest)	19,813 (15.3)	17,347 (15.7)	1635 (13.5)	346 (11.4)	304 (12.9)	181 (11.9)
Smoking, *N* (%)						
Never smoker	100,983 (78.0)	91,519 (82.9)	7189 (59.5)	1163 (38.4)	714 (30.2)	398 (26.1)
Past smoker	15,971 (12.3)	10,740 (9.7)	2963 (24.5)	950 (31.4)	817 (34.6)	501 (32.9)
Current smoker	12,492 (9.7)	8188 (7.4)	1935 (16.0)	913 (30.2)	832 (35.2)	624 (41.0)
Physical activity, times per week, *N* (%)						
0	66,277 (51.2)	59,251 (53.7)	4309 (35.7)	1145 (37.8)	907 (38.4)	665 (43.7)
1–2	36,411 (28.1)	29,291 (26.5)	4742 (39.2)	1069 (35.3)	832 (35.2)	477 (31.3)
3–4	19,147 (14.8)	15,679 (14.2)	2185 (18.1)	577 (19.1)	446 (18.9)	260 (17.1)
≥5	7611 (5.9)	6226 (5.6)	851 (7.0)	235 (7.8)	178 (7.5)	121 (7.9)
Body mass index (kg/m^2^), mean (SD)	23.9 (3.0)	23.9 (3.0)	23.9 (2.8)	24.1 (2.9)	24.3 (2.9)	24.4 (2.9)
Systolic blood pressure (mmHg), mean (SD)	124.2 (15.2)	124.1 (15.3)	123.8 (14.6)	126.1 (14.7)	127.7 (14.5)	128.3 (15.1)
Triglyceride (mg/dL), mean (SD)	132.3 (82.6)	130.7 (80.2)	131.6 (83.0)	149.5 (102.9)	161.8 (116.7)	170.5 (114.6)
Fasting serum glucose (mg/dL), mean (SD)	99.3 (23.8)	99.0 (23.5)	99.6 (23.7)	103.0 (26.8)	104.5 (26.6)	106.1 (29.5)
Total cholesterol (mg/dL), mean (SD)	201.1 (37.6)	201.5 (37.7)	199.4 (36.2)	197.9 (36.5)	197.1 (35.8)	196.7 (38.6)
Charlson Comorbidity Index, *N* (%)						
0	24,821 (19.2)	20,330 (18.4)	2805 (23.2)	731 (24.2)	594 (25.1)	361 (23.7)
1	36,325 (28.1)	30,640 (27.7)	3576 (29.6)	944 (31.2)	709 (30.0)	456 (29.9)
2	30,094 (23.3)	25,830 (23.4)	2803 (23.2)	647 (21.4)	481 (20.4)	333 (21.9)
≥3	38,206 (29.5)	33,647 (30.5)	2903 (24.0)	704 (23.3)	579 (24.5)	373 (24.5)

Abbreviation: SD, standard deviation.

^a^Proxy for socioeconomic status based on the insurance premium of the National Health Insurance Service.

**Table 2:** Hazard ratios of the risk of depression and suicide according to changes in alcohol consumption categorized by the number of glasses per day among initial nondrinkers.

Depression and suicide	Alcohol intake during the third health examination (drinks per day)				
	0	>0–≤1	>1–≤ 2	>2–≤4	>4
Depression					
Total population					
Events	8537	674	154	123	95
Person-years	954,129	105,694	26,525	20,689	13,285
aHR (95% CI)					
Model 1	1.00 (reference)	0.88 (0.82–0.96)	0.90 (0.77–1.06)	0.94 (0.78–1.12)	1.14 (0.93–1.39)
Model 2	1.00 (reference)	0.89 (0.82–0.96)	0.90 (0.77–1.06)	0.94 (0.78–1.13)	1.14 (0.93–1.40)
Model 3	1.00 (reference)	0.91 (0.84–0.98)	0.94 (0.80–1.10)	0.97 (0.81–1.17)	1.18 (0.96–1.45)
Never drinker					
Events	7526	360	35	32	13
Person-years	823,093	52,640	6322	3574	1852
aHR (95% CI)					
Model 1	1.00 (reference)	0.90 (0.81–1.00)	0.79 (0.56–1.10)	1.27 (0.90–1.80)	1.01 (0.59–1.74)
Model 2	1.00 (reference)	0.90 (0.81–1.00)	0.79 (0.56–1.10)	1.27 (0.89–1.79)	1.01 (0.59–1.74)
Model 3	1.00 (reference)	0.91 (0.82–1.02)	0.81 (0.58–1.12)	1.28 (0.90–1.81)	1.01 (0.58–1.74)
** **Former drinker					
Events	1011	314	119	91	82
Person-years	131,037	53,053	20,203	17,115	11,433
aHR (95% CI)					
Model 1	1.00 (reference)	0.90 (0.79–1.02)	0.99 (0.82–1.21)	0.91 (0.73–1.14)	1.23 (0.98–1.55)
Model 2	1.00 (reference)	0.90 (0.79–1.02)	1.00 (0.82–1.21)	0.91 (0.73–1.14)	1.23 (0.98–1.56)
Model 3	1.00 (reference)	0.94 (0.83–1.07)	1.06 (0.87–1.29)	0.97 (0.78–1.21)	1.31 (1.04–1.66)
Suicide					
Total population					
Events	182	25	9	15	7
Person-years	992,788	108,609	27,192	21,197	13,673
aHR (95% CI)					
Model 1	1.00 (reference)	0.98 (0.64–1.50)	1.15 (0.59–2.27)	2.35 (1.37–4.02)	1.63 (0.76–3.49)
Model 2	1.00 (reference)	0.98 (0.64–1.50)	1.11 (0.56–2.18)	2.23 (1.30–3.83)	1.52 (0.71–3.26)
Model 3	1.00 (reference)	0.98 (0.64–1.50)	1.11 (0.56–2.18)	2.25 (1.31–3.87)	1.53 (0.71–3.29)

*Note:* The aHRs were calculated using Cox proportional hazards regression after adjusting multivariate variables. In Model 1, adjustments were made for sociodemographic factors, including age and gender. Model 2 expanded on this by including additional variables such as household income, smoking habits, and levels of physical activity. Model 3 further incorporated body mass index (BMI), systolic blood pressure, fasting blood glucose levels, total cholesterol, and the Charlson Comorbidity Index, building upon the adjustments made in Model 2.

Abbreviations: aHR, adjusted hazard ratio; CI, confidence interval.

**Table 3:** Hazard ratios of the risk of depression and suicide according to the changes in alcohol consumption categorized by the number of days of drinking alcohol per week among initial nondrinkers.

Depression and suicide	Alcohol intake during the third health examination (days per week)				
	0	1	2	3–4	5–7
Depression					
Total population					
Events	8534	560	205	158	126
Person-years	953,269	94,324	33,393	24,361	14,977
aHR (95% CI)					
Model 1	1.00 (reference)	0.86 (0.78–0.93)	0.93 (0.80–1.07)	0.98 (0.83–1.14)	1.08 (0.90–1.29)
Model 2	1.00 (reference)	0.86 (0.79–0.94)	0.93 (0.81–1.07)	0.98 (0.83–1.15)	1.08 (0.90–1.29)
Model 3	1.00 (reference)	0.88 (0.80–0.96)	0.96 (0.83–1.10)	1.02 (0.87–1.19)	1.12 (0.93–1.34)
Never drinker					
Events	7524	307	67	33	35
Person-years	823,692	46,878	9323	4666	3923
aHR (95% CI)					
Model 1	1.00 (reference)	0.88 (0.79–0.99)	0.95 (0.75–1.21)	0.93 (0.66–1.31)	1.05 (0.75–1.46)
Model 2	1.00 (reference)	0.89 (0.79–0.99)	0.95 (0.75–1.21)	0.93 (0.66–1.31)	1.05 (0.75–1.46)
Model 3	1.00 (reference)	0.90 (0.80–1.01)	0.97 (0.76–1.23)	0.95 (0.67–1.34)	1.07 (0.77–1.49)
Former drinker					
Events	1010	253	138	125	91
Person-years	130,577	47,446	24,069	19,695	11,053
aHR (95% CI)					
Model 1	1.00 (reference)	0.85 (0.74–0.98)	0.96 (0.80–1.15)	1.04 (0.86–1.25)	1.12 (0.90–1.39)
Model 2	1.00 (reference)	0.86 (0.74–0.99)	0.96 (0.80–1.16)	1.04 (0.86–1.26)	1.12 (0.90–1.40)
Model 3	1.00 (reference)	0.89 (0.78–1.03)	1.02 (0.85–1.22)	1.11 (0.91–1.35)	1.18 (0.95–1.48)
Suicide					
Total population					
Events	181	22	9	16	10
Person-years	991,919	96,753	34,255	25,008	15,524
aHR (95% CI)					
Model 1	1.00 (reference)	1.03 (0.65–1.61)	0.97 (0.49–1.91)	2.14 (1.27–3.59)	1.70 (0.89–3.24)
Model 2	1.00 (reference)	1.02 (0.65–1.61)	0.95 (0.48–1.87)	2.01 (1.19–3.40)	1.61 (0.84–3.06)
Model 3	1.00 (reference)	1.03 (0.65–1.62)	0.95 (0.48–1.87)	2.03 (1.20–3.43)	1.57 (0.82–3.00)

*Note:* The aHRs were calculated using Cox proportional hazards regression after adjusting multivariate variables. In Model 1, adjustments were made for sociodemographic factors, including age and gender. Model 2 expanded on this by including additional variables such as household income, smoking habits, and levels of physical activity. Model 3 further incorporated body mass index (BMI), systolic blood pressure, fasting blood glucose levels, total cholesterol, and the Charlson Comorbidity Index, building upon the adjustments made in Model 2.

Abbreviations: aHR, adjusted hazard ratio; CI, confidence interval.

**Table 4:** Adjusted hazard ratios for depression and suicide per one drink per day increment and 1 day per week increment in alcohol intake by population subgroups.

Depression and suicide	Drinks per day			Days per week		
	Model 1	Model 2	Model 3	Model 1	Model 2	Model 3
Depression						
Total population						
Events	9583					
Person-years	1,120,323					
aHR (95% CI)	0.98 (0.94–1.01)	0.98 (0.95–1.02)	0.99 (0.96–1.03)	0.98 (0.95–1.02)	0.98 (0.95–1.02)	1.00 (0.97–1.03)
Never drinker						
Events	7966					
Person-years	887,482					
aHR (95% CI)	0.96 (0.90–1.03)	0.96 (0.90–1.03)	0.97 (0.91–1.04)	0.97 (0.92–1.02)	0.97 (0.92–1.02)	0.98 (0.92–1.03)
Former drinker						
Events	1617					
Person-years	232,841					
aHR (95% CI)	1.01 (0.96–1.06)	1.01 (0.97–1.06)	1.03 (0.98–1.08)	1.01 (0.97–1.06)	1.01 (0.97–1.06)	1.03 (0.99–1.08)
Suicide						
Total population						
Events	238					
Person-years	1,163,460					
aHR (95% CI)	1.19 (1.04–1.35)	1.17 (1.02–1.33)	1.17 (1.02–1.34)	1.17 (1.04–1.32)	1.15 (1.02–1.30)	1.15 (1.02–1.30)

*Note:* The aHRs were calculated using Cox proportional hazards regression after adjusting multivariate variables. In Model 1, adjustments were made for sociodemographic factors, including age and gender. Model 2 expanded on this by including additional variables such as household income, smoking habits, and levels of physical activity. Model 3 further incorporated body mass index (BMI), systolic blood pressure, fasting blood glucose levels, total cholesterol, and the Charlson Comorbidity Index, building upon the adjustments made in Model 2.

Abbreviations: aHR, adjusted hazard ratio; CI, confidence interval.

**Table 5:** Stratified analysis of the association of alcohol consumption changes categorized by the number of glasses per day with depression among initial nondrinkers according to subgroups of sex, age, physical activity, smoking, and Charlson Comorbidity Index.

Stratification variables	Alcohol intake during the third health examination (drinks per day)					*p* for interaction
	0	>0–≤1	>1–≤ 2	>2–≤4	>4	
Sex	—	—	—	—	—	0.42
Men						
Events	2103	365	125	109	87	—
Person-years	309,758	67,485	23,235	19,106	12,747	—
aHR (95% CI)	1.00 (reference)	0.93 (0.83–1.04)	0.95 (0.79–1.14)	0.99 (0.82–1.20)	1.17 (0.94–1.45)	—
Women						
Events	6434	309	29	14	8	—
Person-years	644,372	38,208	3290	1583	538	—
aHR (95% CI)	1.00 (reference)	0.90 (0.80–1.01)	0.98 (0.68–1.41)	0.98 (0.58–1.66)	1.65 (0.82–3.31)	—
Age, years	—	—	—	—	—	0.02
<60						
Events	3561	326	74	51	47	—
Person-years	533,542	70,599	17,957	13,699	8533	—
aHR (95% CI)	1.00 (reference)	0.85 (0.76–0.95)	0.88 (0.70–1.12)	0.83 (0.62–1.10)	1.24 (0.92–1.67)	—
≥60						
Events	4976	348	80	72	48	—
Person-years	420,588	35,095	8568	6990	4753	—
aHR (95% CI)	1.00 (reference)	0.96 (0.86–1.08)	0.98 (0.79–1.23)	1.10 (0.87–1.39)	1.09 (0.81–1.45)	—
Physical activity	—	—	—	—	—	0.03
No						
Events	4922	291	75	56	50	—
Person-years	510,412	37,443	9917	7905	5768	—
aHR (95% CI)	1.00 (reference)	0.97 (0.86–1.10)	1.06 (0.84–1.34)	1.03 (0.79–1.35)	1.27 (0.96–1.69)	—
Yes						
Events	3615	383	79	67	45	—
Person-years	443,718	68,250	16,608	12,784	7517	—
aHR (95% CI)	1.00 (reference)	0.87 (0.78–0.97)	0.85 (0.68–1.07)	0.94 (0.74–1.20)	1.10 (0.82–1.48)	—
Income	—	—	—	—	—	0.59
First to second						
Events	5320	428	94	83	66	—
Person-years	597,781	70,474	18,524	14,187	9036	—
aHR (95% CI)	1.00 (reference)	0.91 (0.82–1.01)	0.87 (0.71–1.07)	1.01 (0.81–1.26)	1.27 (0.99–1.63)	—
Third to fourth						
Events	3217	246	60	40	29	—
Person-years	356,348	35,220	8001	6502	4250	—
aHR (95% CI)	1.00 (reference)	0.90 (0.79–1.02)	1.07 (0.82–1.38)	0.91 (0.66–1.25)	1.01 (0.70–1.46)	—
Smoking	—	—	—	—	—	0.08
Never or past smoker						
Events	8045	593	110	85	66	—
Person-years	882,689	898,658	18,486	13,385	7813	—
aHR (95% CI)	1.00 (reference)	0.92 (0.84–1.00)	0.93 (0.77–1.12)	0.99 (0.80–1.23)	1.33 (1.04–1.70)	—
Current smoker						
Events	492	81	44	38	29	—
Person-years	71,441	17,036	8039	7304	5472	—
aHR (95% CI)	1.00 (reference)	0.82 (0.64–1.03)	0.94 (0.69–1.29)	0.92 (0.66–1.28)	0.92 (0.63–1.35)	—
Charlson Comorbidity Index	—	—	—	—	—	0.15
0–1						
Events	2595	234	63	43	38	—
Person-years	447,033	56,408	14,777	11,505	7192	—
aHR (95% CI)	1.00 (reference)	0.87 (0.76–1.00)	0.98 (0.76–1.27)	0.89 (0.65–1.21)	1.25 (0.90–1.73)	—
≥2						
Events	5942	440	91	80	57	—
Person-years	507,096	49,286	11,748	9184	6093	—
aHR (95% CI)	1.00 (reference)	0.91 (0.83–1.01)	0.89 (0.72–1.10)	1.00 (0.80–1.26)	1.11 (0.85–1.44)	—

*Note:* Adjusted hazard ratio calculated by Cox proportional hazards regression after adjustments for age, sex, household income, smoking, physical activity, body mass index, systolic blood pressure, triglyceride, fasting serum glucose, total cholesterol, and Charlson Comorbidity Index.

Abbreviations: aHR, adjusted hazard ratio; CI, confidence interval.

## Data Availability

The dataset was generated in the NHIS repository (https://nhiss.nhis.or.kr/).

## Supporting Information

Supporting Information Table S1 presents the baseline characteristics of never drinkers and former drinkers, categorized by the number of glasses per day. Moreover, Table S2 provides a detailed overview of the participants' baseline characteristics, categorized by the frequency of alcohol consumption per week. Furthermore, Table S3 shows a stratified analysis of the association between alcohol consumption changes (number of glasses per day) and depression among initial nondrinkers, divided into never drinkers and former drinkers, according to subgroups by sex, age, physical activity, income, and the Charlson Comorbidity Index. In addition, Table S4 examines the association of changes in alcohol consumption (frequency per week) with the risk of depression among initial nondrinkers, according to the same subgroups.
