# Supplementary material for: Associations of Changes in Alcohol Consumption on the Risk of Depression/Suicide Among Initial Nondrinkers
Source: Depress Anxiety. 2024 Nov 11;2024:7560390. doi: 10.1155/2024/7560390 (PMC11919164; doi:10.1155/2024/7560390)
Supplement: Supporting Information — Table S1 presents the baseline characteristics of never drinkers and former drinkers, categorized by the number of glasses per day. Moreover, Table S2 provides a detailed overview of the participants' baseline characteristics, categorized by the frequency of alcohol consumption per week. Furthermore, Table S3 shows a stratified analysis of the association between alcohol consumption changes (number of glasses per day) and depression among initial nondrinkers, divided into never drinkers and former drinkers, according to subgroups by sex, age, physical activity, income, and the Charlson Comorbidity Index. In addition, Table S4 examines the association of changes in alcohol consumption (frequency per week) with the risk of depression among initial nondrinkers, according to the same subgroups. [file 7560390.f1.docx]

**Supplementary Table 1.** Descriptive statistics of the never drinkers and former drinkers in the National Health Insurance Service (alcohol consumption categorized by the number of glasses per day)

| **Participant Characteristics** | **Alcohol intake during second health examination (drinks per day)** | | | | | |
| --- | --- | --- | --- | --- | --- | --- |
|  | **Total** | **0** | **>0–≤1** | **>1–≤ 2** | **>2–≤4** | **>4** |
| **Number of participants (%)** |  |  |  |  |  |  |
| Never drinker | 102,721 | 95,338 (92.8) | 6,034 (5.9) | 720 (0.7) | 415 (0.4) | 214 (0.2) |
| Former drinker | 26,725 | 15,109 (56.5) | 6,053 (22.7) | 2,306 (8.6) | 1,948 (7.3) | 1,309 (4.9) |
| **Age, years, mean (SD)** |  |  |  |  |  |  |
| Never drinker | 60.0 (9.0) | 60.2 (9.0) | 57.7 (8.4) | 58.1 (8.7) | 59.0 (8.9) | 59.4 (8.9) |
| Former drinker | 58.6 (8.6) | 59.3 (8.9) | 57.8 (8.3) | 57.5 (8.2) | 57.6 (8.1) | 57.9 (8.4) |
| **Sex, N (%)** |  |  |  |  |  |  |
| **Never drinker** |  |  |  |  |  |  |
| Men | 31,463 (30.6) | 27,142 (28.5) | 3,240 (53.7) | 550 (76.4) | 341 (82.2) | 190 (88.8) |
| Women | 71,258 (69.4) | 68,196 (71.5) | 2,794 (46.3) | 170 (23.6) | 74 (17.8) | 24 (11.2) |
| **Former drinker** |  |  |  |  |  |  |
| Men | 18,011 (67.4) | 8,363 (55.4) | 4,446 (73.5) | 2,094 (90.8) | 1,839 (94.4) | 1,269 (97.0) |
| Women | 8,714 (32.6) | 6,746 (44.7) | 1,607 (27.0) | 212 (9.2) | 109 (5.6) | 40 (3.1) |
| **Household income^a^, N (%)** |  |  |  |  |  |  |
| **Never drinker** |  |  |  |  |  |  |
| 1^st^ | 34,506 (33.6) | 31,852 (33.4) | 2,217 (36.7) | 239 (33.2) | 130 (31.3) | 68 (31.8) |
| 2^nd^ | 29,965 (29.2) | 27,809 (29.2) | 1,740 (28.8) | 220 (30.6) | 128 (30.8) | 68 (31.8) |
| 3^rd^ | 22,144 (21.6) | 20,619 (21.6) | 1,233 (20.4) | 160 (22.2) | 91 (21.9) | 41 (19.2) |
| 4^th^ | 16,106 (15.7) | 15,058 (15.8) | 844 (14.0) | 101 (14.0) | 66 (15.9) | 37 (17.3) |
| **Former drinker** |  |  |  |  |  |  |
| 1^st^ | 9,652 (36.1) | 5,099 (33.8) | 2,385 (39.4) | 948 (41.1) | 760 (39.0) | 460 (35.1) |
| 2^nd^ | 7,872 (29.5) | 4,421 (29.3) | 1,711 (28.3) | 698 (30.3) | 602 (30.9) | 440 (33.6) |
| 3^rd^ | 5,494 (20.6) | 3,300 (21.8) | 1,166 (19.3) | 415 (18.0) | 348 (17.9) | 265 (20.2) |
| 4^th^ | 3,707 (13.9) | 2,289 (15.2) | 791 (13.1) | 245 (10.6) | 238 (12.2) | 144 (11.0) |
| **Smoking, N (%)** |  |  |  |  |  |  |
| **Never drinker** |  |  |  |  |  |  |
| Never smoker | 85,414 (83.2) | 80,821 (84.8) | 4,014 (66.5) | 349 (48.5) | 165 (39.8) | 65 (30.4) |
| Past smoker | 9,857 (9.6) | 8,155 (8.6) | 1,316 (21.8) | 199 (27.6) | 121 (29.2) | 66 (30.8) |
| Current smoker | 7,450 (7.3) | 6,362 (6.7) | 704 (11.7) | 172 (23.9) | 129 (31.1) | 83 (38.8) |
| **Former drinker** |  |  |  |  |  |  |
| Never smoker | 15,569 (58.3) | 10,698 (70.8) | 3,175 (52.5) | 814 (35.3) | 549 (28.2) | 333 (25.4) |
| Past smoker | 6,114 (22.9) | 2,585 (17.1) | 1,647 (27.2) | 751 (32.6) | 696 (35.7) | 435 (33.2) |
| Current smoker | 5,042 (18.9) | 1,826 (12.1) | 1,231 (20.3) | 741 (32.1) | 703 (36.1) | 541 (41.3) |
| **Physical activity, times per week, N (%)** |  |  |  |  |  |  |
| **Never drinker** |  |  |  |  |  |  |
| 0 | 54,138 (52.7) | 51,358 (53.9) | 2,194 (36.4) | 299 (41.5) | 196 (47.2) | 91 (42.5) |
| 1-2 | 28,035 (27.3) | 25,270 (26.5) | 2,357 (39.1) | 220 (30.6) | 120 (28.9) | 68 (31.8) |
| 3-4 | 14,752 (14.4) | 13,467 (14.1) | 1,055 (17.5) | 130 (18.1) | 67 (16.1) | 33 (15.4) |
| ≥5 | 5,796 (5.6) | 5,243 (5.5) | 428 (7.1) | 71 (9.9) | 32 (7.7) | 22 (10.3) |
| **Former drinker** |  |  |  |  |  |  |
| 0 | 12.139 (45.4) | 7,893 (52.2) | 2,115 (34.9) | 846 (36.7) | 711 (36.5) | 574 (43.9) |
| 1-2 | 8,376 (31.3) | 4,021 (26.6) | 2,385 (39.4) | 849 (36.8) | 712 (36.6) | 409 (31.3) |
| 3-4 | 4,395 (16.5) | 2,212 (14.6) | 1,130 (18.7) | 447 (19.4) | 379 (19.5) | 227 (17.3) |
| ≥5 | 1,815 (6.8) | 983 (6.5) | 423 (7.0) | 164 (7.11) | 146 (7.5) | 99 (7.6) |
| **Body mass index, kg/m^2^, mean (SD)** |  |  |  |  |  |  |
| Never drinker | 23.9 (3.0) | 23.9 (3.0) | 23.8 (2.8) | 24.0 (2.9) | 24.5 (2.8) | 24.2 (2.9) |
| Former drinker | 24.1 (2.9) | 24.0 (2.9) | 24.0 (2.7) | 24.2 (2.8) | 24.3 (2.9) | 24.5 (2.9) |
| **Systolic blood pressure, mmHg, mean (SD)** |  |  |  |  |  |  |
| Never drinker | 124.1 (15.3) | 124.1 (15.4) | 123.5 (14.8) | 125.4 (15.1) | 128.1 (15.2) | 126.4 (14.0) |
| Former drinker | 124.8 (14.8) | 124.2 (14.9) | 124.1 (14.5) | 126.3 (14.6) | 127.6 (14.4) | 128.7 (15.3) |
| **Triglyceride, mg/dL, mean (SD)** |  |  |  |  |  |  |
| Never drinker | 130.4 (80.0) | 130.3 (79.5) | 126.7 (77.6) | 141.1 (94.8) | 169.4 (134.7) | 153.9 (103.0) |
| Former drinker | 139.3 (91.9) | 132.9 (84.4) | 136.4 (87.8) | 152.2 (105.2) | 160.2 (112.4) | 173.2 (116.2) |
| **Fasting serum glucose, mg/dL, mean (SD)** |  |  |  |  |  |  |
| Never drinker | 98.8 (23.1) | 98.7 (23.1) | 99.0 (23.2) | 101.4 (23.9) | 104.4 (25.5) | 103.8 (27.5) |
| Former drinker | 101.2 (26.1) | 100.4 (26.1) | 100.2 (24.0) | 103.5 (27.7) | 104.5 (26.8) | 106.5 (29.8) |
| **Total cholesterol, mg/dL mean (SD)** |  |  |  |  |  |  |
| Never drinker | 201.8 (37.6) | 201.9 (37.7) | 200.5 (36.7) | 197.4 (37.5) | 199.0 (35.7) | 191.9 (36.7) |
| Former drinker | 198.3 (37.2) | 198.6 (37.9) | 198.3 (35.7) | 198.1 (36.2) | 196.7 (35.8) | 197.5 (38.9) |
| **Charlson comorbidity index, N (%)** |  |  |  |  |  |  |
| **Never drinker** |  |  |  |  |  |  |
| 0 | 19,123 (18.6) | 17,445 (18.3) | 1,382 (22.9) | 161 (22.4) | 90 (21.7) | 45 (21.0) |
| 1 | 28,598 (27.8) | 26,456 (27.8) | 1,751 (29.0) | 218 (30.3) | 113 (27.2) | 60 (28.0) |
| 2 | 24,115 (23.5) | 22,389 (23.5) | 1,434 (23.8) | 157 (21.8) | 90 (21.7) | 45 (21.0) |
| ≥3 | 30,885 (30.1) | 29,048 (30.5) | 1,467 (24.3) | 184 (25.6) | 122 (29.4) | 64 (29.9) |
| **Former drinker** |  |  |  |  |  |  |
| 0 | 5,698 (21.3) | 2,885 (19.1) | 1,423 (23.5) | 570 (24.7) | 504 (25.8) | 316 (24.1) |
| 1 | 7,727 (28.9) | 4,184 (27.7) | 1,825 (30.2) | 726 (31.5) | 596 (30.6) | 396 (30.3) |
| 2 | 5,979 (22.4) | 3,441 (22.8) | 1,369 (22.6) | 490 (21.3) | 391 (20.1) | 288 (22.0) |
| ≥3 | 7,321 (27.4) | 4,599 (30.4) | 1,436 (23.7) | 520 (22.6) | 457 (23.5) | 309 (23.6) |

SD: standard deviation.

^a^Proxy for socioeconomic status based on the insurance premium of the National Health Insurance Service.

**Supplementary Table 2.** Descriptive statistics of the participants in the National Health Insurance Service (alcohol consumption categorized by the number of days of drinking alcohol per week)

| **Participant Characteristics** | **Alcohol intake during third health examination (days per week)** | | | | | |
| --- | --- | --- | --- | --- | --- | --- |
|  | **Total** | **0** | **1** | **2** | **3-4** | **5-7** |
| **Number of participants (%)** |  |  |  |  |  |  |
| Total study population | 129,446 | 110,350 (85.3) | 10,768 (8.3) | 3,811 (2.9) | 2,787 (2.2) | 1,730 (1.3) |
| Never drinker | 102,721 | 95,293 (92.8) | 5,368 (5.2) | 1,070 (1.0) | 534 (0.5) | 456 (0.4) |
| Former drinker | 26,725 | 15,057 (56.3) | 5,400 (20.2) | 2,741 (10.3) | 2,253 (8.4) | 1,274 (4.8) |
| **Age, years, mean (SD)** |  |  |  |  |  |  |
| Total study population | 59.7 (9.0) | 60.1 (9.0) | 56.9 (7.8) | 57.6 (8.3) | 58.5 (8.5) | 62.7 (9.6) |
| Never drinker | 60.0 (9.0) | 60.2 (9.0) | 57.1 (8.0) | 58.9 (9.0) | 59.9 (9.2) | 62.7 (9.8) |
| Former drinker | 58.6 (8.6) | 59.3 (8.9) | 56.7 (7.6) | 57.1 (8.0) | 58.2 (8.3) | 62.8 (9.6) |
| **Sex, N (%)** |  |  |  |  |  |  |
| **Total study population** |  |  |  |  |  |  |
| Men | 49,474 (38.2) | 35,447 (32.1) | 6,992 (64.9) | 3,104 (81.5) | 2,449 (87.9) | 1,482 (85.7) |
| Women | 79,972 (61.8) | 74,903 (67.9) | 3,776 (35.1) | 707 (18.6) | 338 (12.1) | 248 (14.3) |
| **Never drinker** |  |  |  |  |  |  |
| Men | 31,463 (30.6) | 27,123 (28.5) | 2,926 (54.5) | 707 (66.1) | 394 (73.8) | 313 (68.6) |
| Women | 71,258 (69.4) | 68,170 (71.5) | 2,442 (45.5) | 363 (33.9) | 140 (26.2) | 143 (31.4) |
| **Former drinker** |  |  |  |  |  |  |
| Men | 18,011 (67.4) | 8,324 (55.3) | 4,066 (75.3) | 2,397 (87.5) | 2,055 (91.2) | 1,169 (91.8) |
| Women | 8,714 (32.6) | 6,733 (44.7) | 1,334 (24.7) | 344 (12.6) | 198 (8.8) | 105 (8.2) |
| **Household income^a^, N (%)** |  |  |  |  |  |  |
| **Total study population** |  |  |  |  |  |  |
| 1^st^ (highest) | 44,158 (34.1) | 36,915 (33.5) | 4,254 (39.5) | 1,532 (40.2) | 961 (34.5) | 496 (28.7) |
| 2^nd^ | 37,837 (29.2) | 32,202 (29.2) | 3,053 (28.4) | 1,116 (29.3) | 853 (30.6) | 613 (35.4) |
| 3^rd^ | 27,638 (21.4) | 23,898 (21.7) | 2,050 (19.0) | 718 (18.8) | 596 (21.4) | 376 (21.7) |
| 4^th^ | 19,813 (15.3) | 17,335 (15.7) | 1,411 (13.1) | 445 (11.7) | 377 (13.5) | 245 (14.2) |
| **Never drinker** |  |  |  |  |  |  |
| 1^st^ | 34,506 (33.6) | 31,841 (33.4) | 2,001 (37.3) | 376 (35.1) | 163 (30.5) | 125 (27.4) |
| 2^nd^ | 29,965 (29.2) | 27,796 (29.2) | 1,541 (28.7) | 312 (29.2) | 166 (31.1) | 150 (32.9) |
| 3^rd^ | 22,144 (21.6) | 20,604 (21.6) | 1,081 (20.1) | 227 (21.2) | 127 (23.8) | 105 (23.0) |
| 4^th^ | 16,106 (15.7) | 15,052 (15.8) | 745 (13.9) | 155 (14.5) | 78 (14.6) | 76 (16.7) |
| **Former drinker** |  |  |  |  |  |  |
| 1^st^ | 9,652 (36.1) | 5,074 (33.7) | 2,253 (41.7) | 1,156 (42.2) | 798 (35.4) | 371 (29.1) |
| 2^nd^ | 7,872 (29.5) | 4,406 (29.3) | 1,512 (28.0) | 804 (29.3) | 687 (30.5) | 463 (36.3) |
| 3^rd^ | 5,494 (20.6) | 3,294 (21.9) | 969 (17.9) | 491 (17.9) | 469 (20.8) | 271 (21.3) |
| 4^th^ | 3,707 (13.9) | 2,283 (15.2) | 666 (12.3) | 290 (10.6) | 299 (13.3) | 169 (13.3) |
| **Smoking, N (%)** |  |  |  |  |  |  |
| **Total study population** |  |  |  |  |  |  |
| Never smoker | 100,983 (78.0) | 91,454 (82.9) | 6,203 (57.6) | 1,657 (43.5) | 1,009 (36.2) | 660 (38.2) |
| Past smoker | 15,971 (12.3) | 10,731 (9.7) | 2,708 (25.2) | 1,151 (30.2) | 874 (31.4) | 507 (29.3) |
| Current smoker | 12,492 (9.7) | 8,165 (7.4) | 1,857 (17.3) | 1,003 (26.3) | 904 (32.4) | 563 (32.5) |
| **Never drinker** |  |  |  |  |  |  |
| Never smoker | 85,414 (83.2) | 80,784 (84.8) | 3,515 (65.5) | 602 (56.3) | 279 (52.3) | 234 (51.3) |
| Past smoker | 9,857 (9.6) | 8,153 (8.6) | 1,176 (21.9) | 282 (26.4) | 125 (23.4) | 121 (26.5) |
| Current smoker | 7,450 (7.3) | 6,356 (6.7) | 677 (12.6) | 186 (17.4) | 130 (24.3) | 101 (22.2) |
| **Former drinker** |  |  |  |  |  |  |
| Never smoker | 15,569 (58.3) | 10,670 (70.9) | 2,688 (49.8) | 1,055 (38.5) | 730 (32.4) | 426 (33.4) |
| Past smoker | 6,114 (22.9) | 2,578 (17.1) | 1,532 (28.4) | 869 (31.7) | 749 (33.2) | 386 (30.3) |
| Current smoker | 5,042 (18.9) | 1,809 (12.0) | 1,180 (21.9) | 817 (29.8) | 774 (34.4) | 462 (36.3) |
| **Physical activity, times per week, N (%)** |  |  |  |  |  |  |
| **Total study population** |  |  |  |  |  |  |
| 0 | 66,277 (51.2) | 59,213 (53.7) | 3,721 (34.6) | 1,364 (35.8) | 1,115 (40.0) | 864 (49.9) |
| 1-2 | 36,411 (28.1) | 29,252 (26.5) | 4,364 (40.5) | 1,475 (38.7) | 896 (32.2) | 424 (24.5) |
| 3-4 | 19,147 (14.8) | 15,664 (14.2) | 1,927 (17.9) | 731 (19.2) | 561 (20.1) | 264 (15.3) |
| ≥5 | 7,611 (5.9) | 6,221 (5.6) | 756 (7.0) | 241 (6.3) | 215 (7.7) | 178 (10.3) |
| **Never drinker** |  |  |  |  |  |  |
| 0 | 54,138 (52.7) | 51,339 (53.9) | 1,919 (35.8) | 421 (39.4) | 247 (46.3) | 212 (46.5) |
| 1-2 | 28,035 (27.3) | 25,254 (26.5) | 2,153 (40.1) | 368 (34.4) | 146 (27.3) | 114 (25.0) |
| 3-4 | 14,752 (14.4) | 13,460 (14.1) | 917 (17.1) | 199 (18.6) | 103 (19.3) | 73 (16.0) |
| ≥5 | 5,796 (5.6) | 5,240 (5.5) | 379 (7.1) | 82 (7.7) | 38 (7.1) | 57 (12.5) |
| **Former drinker** |  |  |  |  |  |  |
| 0 | 12,139 (45.4) | 7,874 (52.3) | 1,802 (33.4) | 943 (34.4) | 868 (38.5) | 652 (51.2) |
| 1-2 | 8,376 (31.3) | 3,998 (26.6) | 2,211 (40.9) | 1,107 (40.4) | 750 (33.3) | 310 (24.3) |
| 3-4 | 4,395 (16.5) | 2,204 (14.6) | 1,010 (18.7) | 532 (19.4) | 458 (20.3) | 191 (15.0) |
| ≥5 | 1,815 (6.8) | 981 (6.5) | 377 (7.0) | 159 (5.8) | 177 (7.9) | 121 (9.5) |
| **Body mass index, kg/m^2^, mean (SD)** |  |  |  |  |  |  |
| Total study population | 23.9 (3.0) | 23.9 (3.0) | 24.0 (2.8) | 24.1 (2.8) | 24.3 (2.8) | 23.8 (2.9) |
| Never drinker | 23.9 (3.0) | 23.9 (3.0) | 23.8 (2.8) | 23.9 (2.9) | 24.0 (2.9) | 23.9 (2.9) |
| Former drinker | 24.1 (2.9) | 24.0 (2.9) | 24.1 (2.7) | 24.2 (2.7) | 24.3 (2.8) | 23.8 (3.0) |
| **Systolic blood pressure, mmHg, mean (SD)** |  |  |  |  |  |  |
| Total study population | 124.2 (15.2) | 124.1 (15.3) | 123.5 (14.5) | 125.8 (14.8) | 127.6 (14.8) | 128.3 (15.2) |
| Never drinker | 124.1 (15.3) | 124.1 (15.4) | 123.2 (14.6) | 125.3 (15.6) | 126.7 (15.2) | 126.8 (14.8) |
| Former drinker | 124.8 (14.8) | 124.2 (14.9) | 123.8 (14.3) | 126.0 (14.5) | 127.8 (14.7) | 128.9 (15.3) |
| **Triglyceride, mg/dL, mean (SD)** |  |  |  |  |  |  |
| Total study population | 132.3 (82.6) | 130.7 (80.2) | 133.5 (84.1) | 145.6 (100.0) | 157.4 (113.7) | 153.9 (106.3) |
| Never drinker | 130.4 (79.9) | 130.3 (79.5) | 128.3 (79.1) | 134.1 (91.3) | 150.5 (112.2) | 138.3 (97.4) |
| Former drinker | 139.3 (91.9) | 133.0 (84.5) | 138.6 (88.5) | 150.2 (102.9) | 159.1 (114.0) | 159.5 (108.8) |
| **Fasting serum glucose, mg/dL, mean (SD)** |  |  |  |  |  |  |
| Total study population | 99.3 (23.8) | 99.0 (23.5) | 99.7 (24.1) | 102.2 (25.2) | 104.2 (27.2) | 104.6 (27.7) |
| Never drinker | 98.8 (23.1) | 98.7 (23.1) | 98.9 (23.6) | 100.8 (22.6) | 102.6 (24.5) | 102.9 (25.1) |
| Former drinker | 101.2 (26.1) | 100.3 (23.1) | 100.5 (24.5) | 102.7 (26.1) | 104.6 (27.8) | 105.3 (28.5) |
| **Total cholesterol, mg/dL mean (SD)** |  |  |  |  |  |  |
| Total study population | 201.1 (37.6) | 201.5 (37.7) | 199.5 (36.2) | 198.5 (36.7) | 197.3 (35.9) | 196.0 (38.4) |
| Never drinker | 201.8 (37.6) | 201.9 (37.7) | 200.7 (36.8) | 199.1 (36.5) | 196.5 (36.2) | 195.8 (38.7) |
| Former drinker | 198.3 (37.2) | 198.6 (37.9) | 198.3 (35.5) | 198.2 (36.8) | 197.5 (35.8) | 196.1 (38.3) |
| **Charlson comorbidity index, N (%)** |  |  |  |  |  |  |
| **Total study population** |  |  |  |  |  |  |
| 0 | 24,821 (19.2) | 20,303 (18.4) | 2,580 (24.0) | 905 (23.8) | 682 (24.5) | 351 (20.3) |
| 1 | 36,325 (28.1) | 30,610 (27.7) | 3,232 (30.0) | 1,177 (30.9) | 826 (29.6) | 480 (27.8) |
| 2 | 30,094 (23.3) | 25,810 (23.4) | 2,477 (23.0) | 839 (22.0) | 608 (21.8) | 360 (20.8) |
| ≥3 | 38,206 (29.5) | 33,627 (30.5) | 2,479 (23.0) | 890 (23.4) | 671 (24.1) | 539 (31.2) |
| **Never drinker** |  |  |  |  |  |  |
| 0 | 19,123 (18.6) | 17,432 (18.3) | 1,254 (23.4) | 236 (22.1) | 113 (21.2) | 88 (19.3) |
| 1 | 28,598 (27.8) | 26,443 (27.8) | 1,584 (29.5) | 310 (29.0) | 146 (27.3) | 115 (25.2) |
| 2 | 24,115 (23.5) | 22,380 (23.5) | 1,268 (23.6) | 244 (22.8) | 118 (22.1) | 105 (23.0) |
| ≥3 | 30,885 (30.1) | 29,038 (30.5) | 1,262 (23.5) | 280 (26.2) | 157 (29.4) | 148 (32.5) |
| **Former drinker** |  |  |  |  |  |  |
| 0 | 5,698 (21.3) | 2,871 (19.1) | 1,326 (24.6) | 669 (24.4) | 569 (25.3) | 263 (20.6) |
| 1 | 7,727 (28.9) | 4,167 (27.7) | 1,648 (30.5) | 867 (31.6) | 680 (30.2) | 365 (28.7) |
| 2 | 5,979 (22.4) | 3,430 (22.8) | 1,209 (22.4) | 595 (21.7) | 490 (21.8) | 255 (20.0) |
| ≥3 | 7,321 (27.4) | 4,589 (30.5) | 1,217 (22.5) | 610 (22.3) | 514 (22.8) | 391 (30.7) |

SD: standard deviation.

^a^Proxy for socioeconomic status based on the insurance premium of the National Health Insurance Service.

**Supplementary Table 3.** Stratified analysis of the association of alcohol consumption changes categorized by the number of glasses per day with **depression** among initial non-drinkers, divided into never drinkers and former drinkers, according to subgroups of sex, age, physical activity, smoking, and Charlson comorbidity index

| **Stratification Variables** | **Alcohol intake during second health examination**  **(drinks per day)** | | | | | ***p* for interaction** |
| --- | --- | --- | --- | --- | --- | --- |
|  | **0** | **>0–≤1** | **>1–≤ 2** | **>2–≤4** | **>4** |  |
| **Sex** |  |  |  |  |  |  |
| **Never drinker** |  |  |  |  |  | 0.68 |
| Men |  |  |  |  |  |  |
| Events | 1,638 | 167 | 23 | 24 | 9 |  |
| Person-years | 236,730 | 28,389 | 4,849 | 2,949 | 1,656 |  |
| aHR (95% CI) | 1.00 (reference) | 0.99  (0.84-1.16) | 0.78  (0.51-1.17) | 1.27  (0.85-1.90) | 0.83  (0.43-1.60) |  |
| Women |  |  |  |  |  |  |
| Events | 5,888 | 193 | 12 | 8 | 4 |  |
| Person-years | 586,363 | 24,251 | 1,473 | 626 | 196 |  |
| aHR (95% CI) | 1.00 (reference) | 0.87  (0.75-1.00) | 0.91  (0.51-1.59) | 1.34  (0.67-2.69) | 2.12  (0.80-5.67) |  |
| **Former drinker** |  |  |  |  |  | 0.86 |
| Men |  |  |  |  |  |  |
| Events | 465 | 198 | 102 | 85 | 78 |  |
| Person-years | 73,028 | 39,096 | 18,386 | 16,158 | 11,091 |  |
| aHR (95% CI) | 1.00 (reference) | 0.93  (0.78-1.10) | 1.07  (0.86-1.33) | 1.00  (0.79-1.26) | 1.31  (1.02-1.67) |  |
| Women |  |  |  |  |  |  |
| Events | 546 | 116 | 17 | 6 | 4 |  |
| Person-years | 58,008 | 13,957 | 1,817 | 957 | 342 |  |
| aHR (95% CI) | 1.00 (reference) | 0.97  (0.79-1.18) | 1.07  (0.66-1.73) | 0.74  (0.33-1.65) | 1.43  (0.53-3.86) |  |
| **Age, years** |  |  |  |  |  |  |
| **Never drinker** |  |  |  |  |  | 0.69 |
| <60 |  |  |  |  |  |  |
| Events | 3,104 | 184 | 17 | 17 | 4 |  |
| Person-years | 455,098 | 35,284 | 4,075 | 2,125 | 1,087 |  |
| aHR (95% CI) | 1.00  (reference) | 0.89  (0.77-1.03) | 0.79  (0.49-1.28) | 1.55  (0.96-2.50) | 0.76  (0.29-2.04) |  |
| ≥60 |  |  |  |  |  |  |
| Events | 4,422 | 176 | 18 | 15 | 9 |  |
| Person-years | 367,995 | 17,357 | 2,247 | 1,449 | 765 |  |
| aHR (95% CI) | 1.00  (reference) | 0.94  (0.81-1.09) | 0.80  (0.50-1.27) | 1.02  (0.61-1.70) | 1.16  (0.60-2.24) |  |
| **Former Drinker** |  |  |  |  |  | 0.17 |
| <60 |  |  |  |  |  |  |
| Events | 457 | 142 | 57 | 34 | 43 |  |
| Person-years | 78,444 | 35,315 | 13,882 | 11,574 | 7,445 |  |
| aHR (95% CI) | 1.00  (reference) | 0.83  (0.69-1.01) | 0.99  (0.74-1.32) | 0.74  (0.51-1.06) | 1.46  (1.04-2.03) |  |
| ≥60 |  |  |  |  |  |  |
| Events | 554 | 172 | 62 | 57 | 39 |  |
| Person-years | 52,592 | 17,738 | 6,321 | 5,541 | 3,988 |  |
| aHR (95% CI) | 1.00  (reference) | 1.03  (0.86-1.23) | 1.11  (0.85-1.46) | 1.17  (0.89-1.56) | 1.13  (0.81-1.58) |  |
| **Physical activity** |  |  |  |  |  |  |
| **Never drinker** |  |  |  |  |  | 0.77 |
| No |  |  |  |  |  |  |
| Events | 4,372 | 154 | 18 | 13 | 7 |  |
| Person-years | 442,077 | 19,042 | 2,606 | 1,709 | 774 |  |
| aHR (95% CI) | 1.00  (reference) | 0.95  (0.81-1.11) | 0.89  (0.56-1.42) | 0.99  (0.57-1.71) | 1.13  (0.54-2.37) |  |
| Yes |  |  |  |  |  |  |
| Events | 3,154 | 206 | 17 | 19 | 6 |  |
| Person-years | 381,016 | 33,599 | 3,717 | 1,866 | 1,078 |  |
| aHR (95% CI) | 1.00  (reference) | 0.90  (0.78-1.04) | 0.74  (0.46-1.19) | 1.58  (1.01-2.49) | 0.91  (0.41-2.04) |  |
| **Former drinker** |  |  |  |  |  | 0.02 |
| No |  |  |  |  |  |  |
| Events | 550 | 137 | 57 | 43 | 43 |  |
| Person-years | 68,335 | 18,402 | 7,311 | 6,196 | 4,994 |  |
| aHR (95% CI) | 1.00  (reference) | 1.09  (0.90-1.32) | 1.29  (0.97-1.70) | 1.19  (0.86-1.65) | 1.52  (1.10-2.10) |  |
| Yes |  |  |  |  |  |  |
| Events | 461 | 177 | 62 | 48 | 39 |  |
| Person-years | 62,702 | 34,652 | 12,892 | 10,919 | 6,439 |  |
| aHR (95% CI) | 1.00  (reference) | 0.83  (0.70-0.99) | 0.89  (0.68-1.18) | 0.82  (0.60-1.11) | 1.14  (0.81-1.60) |  |
| **Income** |  |  |  |  |  |  |
| **Never drinker** |  |  |  |  |  | 0.62 |
| 1^st-^2^nd^ |  |  |  |  |  |  |
| Events | 4,708 | 220 | 23 | 23 | 9 |  |
| Person-years | 515,108 | 34,548 | 4,023 | 2,207 | 1,169 |  |
| aHR (95% CI) | 1.00  (reference) | 0.89  (0.78-1.02) | 0.85  (0.57-1.29) | 1.52  (1.01-2.30) | 1.14  (0.59-2.19) |  |
| 3^rd-^4^th^ |  |  |  |  |  |  |
| Events | 2,818 | 140 | 12 | 9 | 4 |  |
| Person-years | 307,984 | 18,093 | 2,299 | 1,367 | 683 |  |
| aHR (95% CI) | 1.00  (reference) | 0.95  (0.80-1.13) | 0.72  (0.41-1.27) | 0.89  (0.46-1.72) | 0.81  (0.30-2.17) |  |
| **Former drinker** |  |  |  |  |  | 0.87 |
| 1^st-^2^nd^ |  |  |  |  |  |  |
| Events | 612 | 208 | 71 | 60 | 57 |  |
| Person-years | 82,672 | 35,926 | 14,501 | 11,980 | 7,866 |  |
| aHR (95% CI) | 1.00  (reference) | 0.99  (0.84-1.16) | 0.97  (0.75-1.25) | 0.99  (0.75-1.30) | 1.44  (1.08-1.91) |  |
| 3^rd-^4^th^ |  |  |  |  |  |  |
| Events | 399 | 106 | 48 | 31 | 25 |  |
| Person-years | 48,364 | 17,127 | 5,702 | 5,135 | 3,567 |  |
| aHR (95% CI) | 1.00  (reference) | 0.85  (0.69-1.06) | 1.25  (0.92-1.70) | 0.94  (0.64-1.37) | 1.08  (0.71-1.64) |  |
| **Smoking** |  |  |  |  |  |  |
| **Never drinker** |  |  |  |  |  | 0.20 |
| Never or past smoker |  |  |  |  |  |  |
| Events | 7,143 | 329 | 20 | 21 | 7 |  |
| Person-years | 767,556 | 46,458 | 4,842 | 2,460 | 1,142 |  |
| aHR (95% CI) | 1.00 (reference) | 0.92  (0.82-1.03) | 0.59  (0.38-0.91) | 1.15  (0.75-1.76) | 0.82  (0.39-1.72) |  |
| Current smoker |  |  |  |  |  |  |
| Events | 383 | 31 | 15 | 11 | 6 |  |
| Person-years | 55,537 | 6,183 | 1,481 | 1,115 | 710 |  |
| aHR (95% CI) | 1.00 (reference) | 0.87  (0.60-1.25) | 1.61  (0.96-2.70) | 1.58  (0.86-2.88) | 1.40  (0.62-3.13) |  |
| **Former drinker** |  |  |  |  |  | 0.01 |
| Never or past smoker |  |  |  |  |  |  |
| Events | 902 | 264 | 90 | 64 | 59 |  |
| Person-years | 115,133 | 42,200 | 13,644 | 10,926 | 6,671 |  |
| aHR (95% CI) | 1.00 (reference) | 0.97  (0.85-1.12) | 1.17  (0.93-1.46) | 1.04  (0.80-1.35) | 1.57  (1.20-2.07) |  |
| Current smoker |  |  |  |  |  |  |
| Events | 109 | 50 | 29 | 27 | 23 |  |
| Person-years | 15,904 | 10,853 | 6,559 | 6,189 | 4,762 |  |
| aHR (95% CI) | 1.00 (reference) | 0.76  (0.54-1.06) | 0.75  (0.49-1.13) | 0.77  (0.50-1.18) | 0.84  (0.53-1.32) |  |
| **Charlson comorbidity index** |  |  |  |  |  |  |
| **Never drinker** |  |  |  |  |  | 0.61 |
| 0-1 |  |  |  |  |  |  |
| Events | 2,264 | 118 | 18 | 13 | 7 |  |
| Person-years | 384,922 | 27,653 | 3,329 | 1,753 | 903 |  |
| aHR (95% CI) | 1.00 (reference) | 0.86  (0.71-1.04) | 1.17  (0.73-1.86) | 1.65  (0.95-2.85) | 1.66  (0.79-3.49) |  |
| ≥2 |  |  |  |  |  |  |
| Events | 5,262 | 242 | 17 | 19 | 6 |  |
| Person-years | 438,171 | 24,987 | 2,993 | 1,821 | 949 |  |
| aHR (95% CI) | 1.00 (reference) | 0.94  (0.82-1.07) | 0.59  (0.37-0.95) | 1.07  (0.68-1.68) | 0.69  (0.31-1.54) |  |
| **Former drinker** |  |  |  |  |  | 0.04 |
| 0-1 |  |  |  |  |  |  |
| Events | 331 | 116 | 45 | 30 | 31 |  |
| Person-years | 62,112 | 28,755 | 11,448 | 9,751 | 6,289 |  |
| aHR (95% CI) | 1.00 (reference) | 0.85  (0.69-1.06) | 0.90  (0.65-1.24) | 0.72  (0.49-1.06) | 1.13  (0.77-1.66) |  |
| ≥2 |  |  |  |  |  |  |
| Events | 680 | 198 | 74 | 61 | 51 |  |
| Person-years | 68,925 | 24,299 | 8,755 | 7,363 | 5,145 |  |
| aHR (95% CI) | 1.00 (reference) | 0.95  (0.81-1.12) | 1.12  (0.87-1.44) | 1.11  (0.84-1.45) | 1.35  (1.00-1.82) |  |

The adjusted hazard ratio was calculated using Cox proportional hazards regression after adjusting age, sex, household income, smoking, physical activity, body mass index, systolic blood pressure, triglyceride, fasting serum glucose, total cholesterol and Charlson comorbidity index.

**Supplementary Table 4.** Stratified analysis of the association of alcohol consumption changes categorized by the number of days of drinking alcohol per week with **depression** among initial non-drinkers according to subgroups of sex, age, physical activity, smoking, and Charlson comorbidity index

| **Stratification Variables** | **Alcohol intake during second health examination**  **(days per week)** | | | | | ***p* for interaction** |
| --- | --- | --- | --- | --- | --- | --- |
|  | **0** | **1** | **2** | **3-4** | **5-7** |  |
| **Sex** |  |  |  |  |  |  |
| **Total Population** |  |  |  |  |  | 0.85 |
| Men |  |  |  |  |  |  |
| Events | 2,102 | 302 | 147 | 139 | 99 |  |
| Person-years | 309,238 | 61,516 | 27,287 | 21,408 | 12,883 |  |
| aHR (95% CI) | 1.00 (reference) | 0.89  (0.79-1.01) | 0.95  (0.80-1.12) | 1.11  (0.93-1.32) | 1.06  (0.86-1.29) |  |
| Women |  |  |  |  |  |  |
| Events | 6,432 | 258 | 58 | 19 | 27 |  |
| Person-years | 644,031 | 32,085 | 6,106 | 2,953 | 2,094 |  |
| aHR (95% CI) | 1.00 (reference) | 0.88  (0.78-1.00) | 1.04  (0.80-1.35) | 0.68  (0.43-1.06) | 1.29  (0.89-1.89) |  |
| **Never drinker** |  |  |  |  |  | 0.86 |
| Men |  |  |  |  |  |  |
| Events | 1,637 | 143 | 35 | 26 | 20 |  |
| Person-years | 236,560 | 25,666 | 6,193 | 3,435 | 2,718 |  |
| aHR (95% CI) | 1.00 (reference) | 0.97  (0.82-1.16) | 0.89  (0.64-1.25) | 1.13  (0.77-1.67) | 0.98  (0.63-1.52) |  |
| Women |  |  |  |  |  |  |
| Events | 5,887 | 164 | 32 | 7 | 15 |  |
| Person-years | 586,132 | 21,211 | 3,131 | 1,231 | 1,205 |  |
| aHR (95% CI) | 1.00 (reference) | 0.86  (0.73-1.00) | 1.08  (0.76-1.53) | 0.60  (0.28-1.25) | 1.20  (0.72-1.99) |  |
| **Former drinker** |  |  |  |  |  | 0.95 |
| Men |  |  |  |  |  |  |
| Events | 465 | 159 | 112 | 113 | 79 |  |
| Person-years | 72,677 | 35,850 | 21,094 | 17,973 | 10,165 |  |
| aHR (95% CI) | 1.00 (reference) | 0.90  (0.73-1.11) | 1.03  (0.73-1.43) | 1.12  (0.72-1.74) | 1.20  (0.69-2.09) |  |
| Women |  |  |  |  |  |  |
| Events | 545 | 94 | 26 | 12 | 12 |  |
| Person-years | 57,899 | 11,596 | 2,975 | 1,722 | 889 |  |
| aHR (95% CI) | 1.00 (reference) | 0.95  (0.77-1.19) | 1.01  (0.68-1.50) | 0.76  (0.43-1.36) | 1.48  (0.83-2.62) |  |
| **Age, years** |  |  |  |  |  |  |
| **Total Population** |  |  |  |  |  | 0.11 |
| <60 |  |  |  |  |  |  |
| Events | 3,561 | 299 | 98 | 67 | 34 |  |
| Person-years | 532,903 | 67,007 | 22,614 | 15,335 | 6,469 |  |
| aHR (95% CI) | 1.00  (reference) | 0.83  (0.74-0.94) | 0.90  (0.73-1.11) | 0.93  (0.73-1.19) | 1.07  (0.76-1.50) |  |
| ≥60 |  |  |  |  |  |  |
| Events | 4,973 | 261 | 107 | 91 | 92 |  |
| Person-years | 420,366 | 27,317 | 10,778 | 9,026 | 8,507 |  |
| aHR (95% CI) | 1.00  (reference) | 0.93  (0.82-1.05) | 1.01  (0.83-1.22) | 1.07  (0.87-1.32) | 1.12  (0.91-1.39) |  |
| **Never drinker** |  |  |  |  |  | 0.88 |
| <60 |  |  |  |  |  |  |
| Events | 3,104 | 165 | 36 | 11 | 10 |  |
| Person-years | 454,810 | 32,887 | 5,650 | 2,619 | 1,703 |  |
| aHR (95% CI) | 1.00  (reference) | 0.86  (0.74-1.01) | 1.15  (0.83-1.60) | 0.77  (0.43-1.40) | 1.03  (0.55-1.92) |  |
| ≥60 |  |  |  |  |  |  |
| Events | 4,420 | 142 | 31 | 22 | 25 |  |
| Person-years | 367,882 | 13,991 | 3,673 | 2,047 | 2,220 |  |
| aHR (95% CI) | 1.00  (reference) | 0.94  (0.79-1.11) | 0.81  (0.57-1.15) | 1.05  (0.69-1.60) | 1.10  (0.74-1.62) |  |
| **Former Drinker** |  |  |  |  |  | 0.35 |
| <60 |  |  |  |  |  |  |
| Events | 457 | 134 | 62 | 56 | 24 |  |
| Person-years | 78,093 | 34,120 | 16,965 | 12,716 | 4,766 |  |
| aHR (95% CI) | 1.00  (reference) | 0.82  (0.68-1.00) | 0.85  (0.65-1.13) | 1.05  (0.79-1.41) | 1.16  (0.76-1.77) |  |
| ≥60 |  |  |  |  |  |  |
| Events | 553 | 119 | 76 | 69 | 67 |  |
| Person-years | 52,483 | 13,326 | 7,105 | 6,979 | 6,287 |  |
| aHR (95% CI) | 1.00  (reference) | 0.95  (0.78-1.17) | 1.17  (0.92-1.50) | 1.13  (0.87-1.46) | 1.20  (0.92-1.56) |  |
| **Physical activity** |  |  |  |  |  |  |
| **Total Population** |  |  |  |  |  | 0.13 |
| No |  |  |  |  |  |  |
| Events | 4,921 | 238 | 94 | 75 | 66 |  |
| Person-years | 510,072 | 32,358 | 11,827 | 9,709 | 7,478 |  |
| aHR (95% CI) | 1.00  (reference) | 0.95  (0.83-1.09) | 1.07  (0.87-1.32) | 1.09  (0.86-1.37) | 1.11  (0.86-1.41) |  |
| Yes |  |  |  |  |  |  |
| Events | 3,613 | 322 | 111 | 83 | 60 |  |
| Person-years | 443,196 | 61,965 | 21,565 | 14,652 | 7,499 |  |
| aHR (95% CI) | 1.00  (reference) | 0.84  (0.75-0.94) | 0.89  (0.73-1.08) | 0.97  (0.77-1.21) | 1.11  (0.86-1.44) |  |
| **Never drinker** |  |  |  |  |  | 0.63 |
| No |  |  |  |  |  |  |
| Events | 4,371 | 128 | 34 | 15 | 16 |  |
| Person-years | 441,908 | 16,675 | 3,628 | 2,165 | 1,831 |  |
| aHR (95% CI) | 1.00  (reference) | 0.92  (0.77-1.10) | 1.14  (0.81-1.60) | 0.85  (0.51-1.41) | 0.97  (0.59-1.58) |  |
| Yes |  |  |  |  |  |  |
| Events | 3,153 | 179 | 33 | 18 | 19 |  |
| Person-years | 380,784 | 30,202 | 5,695 | 2,501 | 2,093 |  |
| aHR (95% CI) | 1.00  (reference) | 0.90  (0.77-1.05) | 0.84  (0.60-1.19) | 1.04  (0.65-1.65) | 1.16  (0.74-1.82) |  |
| **Former drinker** |  |  |  |  |  | 0.12 |
| No |  |  |  |  |  |  |
| Events | 550 | 110 | 60 | 60 | 50 |  |
| Person-years | 68,164 | 15,683 | 8,200 | 7,544 | 5,647 |  |
| aHR (95% CI) | 1.00  (reference) | 1.08  (0.88-1.33) | 1.16  (0.88-1.52) | 1.32  (1.00-1.74) | 1.30  (0.96-1.75) |  |
| Yes |  |  |  |  |  |  |
| Events | 460 | 143 | 78 | 65 | 41 |  |
| Person-years | 62,413 | 31,763 | 15,870 | 12,151 | 5,406 |  |
| aHR (95% CI) | 1.00  (reference) | 0.77  (0.64-0.94) | 0.92  (0.72-1.18) | 0.95  (0.72-1.24) | 1.05  (0.76-1.46) |  |
| **Income** |  |  |  |  |  |  |
| **Total population** |  |  |  |  |  | 0.66 |
| 1^st^-2^nd^ |  |  |  |  |  |  |
| Events | 5,317 | 356 | 124 | 107 | 87 |  |
| Person-years | 597,217 | 64,097 | 23,286 | 15,820 | 9,581 |  |
| aHR (95% CI) | 1.00  (reference) | 0.87  (0.78-0.97) | 0.88  (0.74-1.06) | 1.12  (0.92-1.36) | 1.24  (1.00-1.53) |  |
| 3^rd^-4^th^ |  |  |  |  |  |  |
| Events | 3,217 | 204 | 81 | 51 | 39 |  |
| Person-years | 356,052 | 30,226 | 10,106 | 8,541 | 5,396 |  |
| aHR (95% CI) | 1.00 (reference) | 0.88  (0.77-1.02) | 1.10  (0.88-1.37) | 0.85  (0.64-1.12) | 0.92  (0.67-1.26) |  |
| **Never drinker** |  |  |  |  |  | 0.92 |
| 1^st^-2^nd^ |  |  |  |  |  |  |
| Events | 4,706 | 189 | 40 | 25 | 23 |  |
| Person-years | 514,897 | 30,966 | 6,001 | 2,841 | 2,351 |  |
| aHR (95% CI) | 1.00  (reference) | 0.88  (0.76-1.02) | 0.92  (0.68-1.26) | 1.21  (0.81-1.79) | 1.18  (0.78-1.78) |  |
| 3^rd^-4^th^ |  |  |  |  |  |  |
| Events | 2,818 | 118 | 27 | 8 | 12 |  |
| Person-years | 307,795 | 15,912 | 3,322 | 1,825 | 1,572 |  |
| aHR (95% CI) | 1.00  (reference) | 0.93  (0.77-1.12) | 1.03  (0.71-1.51) | 0.56  (0.28-1.13) | 0.90  (0.51-1.59) |  |
| **Former drinker** |  |  |  |  |  | 0.32 |
| 1^st^-2^nd^ |  |  |  |  |  |  |
| Events | 611 | 167 | 84 | 82 | 64 |  |
| Person-years | 82,320 | 33,131 | 17,285 | 12,979 | 7,230 |  |
| aHR (95% CI) | 1.00  (reference) | 0.92  (0.77-1.10) | 0.95  (0.75-1.20) | 1.20  (0.95-1.53) | 1.34  (1.03-1.76) |  |
| 3^rd^-4^th^ |  |  |  |  |  |  |
| Events | 399 | 86 | 54 | 43 | 27 |  |
| Person-years | 48,256 | 14,315 | 6,784 | 6,716 | 3,823 |  |
| aHR (95% CI) | 1.00  (reference) | 0.86  (0.68-1.08) | 1.15  (0.86-1.55) | 0.95  (0.69-1.32) | 0.92  (0.62-1.37) |  |
| **Smoking** |  |  |  |  |  |  |
| **Total population** |  |  |  |  |  | 0.10 |
| Never or past smoker |  |  |  |  |  |  |
| Events | 8,042 | 486 | 161 | 114 | 96 |  |
| Person-years | 882,035 | 77,968 | 24,541 | 16,423 | 10,065 |  |
| aHR (95% CI) | 1.00 (reference) | 0.89  (0.81-0.97) | 0.97  (0.83-1.14) | 1.03  (0.86-1.24) | 1.20  (0.98-1.47) |  |
| Current smoker |  |  |  |  |  |  |
| Events | 492 | 74 | 44 | 44 | 30 |  |
| Person-years | 71,234 | 16,356 | 8,852 | 7,938 | 4,912 |  |
| aHR (95% CI) | 1.00  (reference) | 0.81  (0.63-1.03) | 0.88  (0.65-1.21) | 0.96  (0.71-1.32) | 0.90  (0.62-1.31) |  |
| **Never drinker** |  |  |  |  |  | 0.44 |
| Never or past smoker |  |  |  |  |  |  |
| Events | 7,141 | 275 | 55 | 22 | 27 |  |
| Person-years | 767,209 | 40,947 | 7,702 | 3,540 | 3,059 |  |
| aHR (95% CI) | 1.00 (reference) | 0.89  (0.79-1.01) | 0.93  (0.71-1.21) | 0.80  (0.53-1.22) | 1.00  (0.69-1.47) |  |
| Current smoker |  |  |  |  |  |  |
| Events | 383 | 32 | 12 | 11 | 8 |  |
| Person-years | 55,483 | 5,930 | 1,621 | 1,126 | 864 |  |
| aHR (95% CI) | 1.00 (reference) | 0.97  (0.67-1.40) | 1.19  (0.67-2.12) | 1.43  (0.78-2.61) | 1.34  (0.67-2.71) |  |
| **Former drinker** |  |  |  |  |  | 0.02 |
| Never or past smoker |  |  |  |  |  |  |
| Events | 901 | 211 | 106 | 92 | 69 |  |
| Person-years | 114,825 | 37,021 | 16,839 | 12,883 | 7,005 |  |
| aHR (95% CI) | 1.00 (reference) | 0.93  (0.80-1.09) | 1.08  (0.88-1.33) | 1.20  (0.96-1.49) | 1.35  (1.05-1.74) |  |
| Current smoker |  |  |  |  |  |  |
| Events | 109 | 42 | 32 | 33 | 22 |  |
| Person-years | 15,751 | 10,425 | 7,231 | 6,812 | 4,048 |  |
| aHR (95% CI) | 1.00 (reference) | 0.68  (0.48-0.98) | 0.77  (0.52-1.15) | 0.86  (0.58-1.28) | 0.78  (0.49-1.24) |  |
| **Charlson comorbidity index** |  |  |  |  |  |  |
| **Total population** |  |  |  |  |  | 0.20 |
| 0-1 |  |  |  |  |  |  |
| Events | 2,594 | 203 | 76 | 65 | 35 |  |
| Person-years | 446,523 | 51,408 | 18,388 | 13,265 | 7,331 |  |
| aHR (95% CI) | 1.00  (reference) | 0.85  (0.74-0.99) | 0.94  (0.74-1.18) | 1.11  (0.86-1.43) | 0.92  (0.66-1.29) |  |
| ≥2 |  |  |  |  |  |  |
| Events | 5,940 | 357 | 129 | 93 | 91 |  |
| Person-years | 506,746 | 42,916 | 15,004 | 11,096 | 7,646 |  |
| aHR (95% CI) | 1.00 (reference) | 0.88  (0.79-0.98) | 0.95  (0.80-1.14) | 0.93  (0.76-1.15) | 1.19  (0.97-1.47) |  |
| **Never drinker** |  |  |  |  |  | 0.51 |
| 0-1 |  |  |  |  |  |  |
| Events | 2,263 | 107 | 24 | 15 | 11 |  |
| Person-years | 384,690 | 25,058 | 4,786 | 2,262 | 1,765 |  |
| aHR (95% CI) | 1.00 (reference) | 0.88  (0.72-1.07) | 1.04  (0.69-1.56) | 1.35  (0.81-2.24) | 1.10  (0.61-2.00) |  |
| ≥2 |  |  |  |  |  |  |
| Events | 5,261 | 200 | 43 | 18 | 24 |  |
| Person-years | 438,002 | 21,819 | 4,538 | 2,404 | 2,159 |  |
| aHR (95% CI) | 1.00 (reference) | 0.90  (0.78-1.04) | 0.92  (0.68-1.24) | 0.74  (0.47-1.18) | 1.04  (0.69-1.55) |  |
| **Former drinker** |  |  |  |  |  | 0.03 |
| 0-1 |  |  |  |  |  |  |
| Events | 331 | 96 | 52 | 50 | 24 |  |
| Person-years | 61,833 | 26,349 | 13,603 | 11,003 | 5,566 |  |
| aHR (95% CI) | 1.00 (reference) | 0.82  (0.65-1.03) | 0.88  (0.65-1.19) | 1.00  (0.74-1.37) | 0.79  (0.52-1.21) |  |
| ≥2 |  |  |  |  |  |  |
| Events | 679 | 157 | 86 | 75 | 67 |  |
| Person-years | 68,744 | 21,097 | 10,467 | 8,692 | 5,487 |  |
| aHR (95% CI) | 1.00 (reference) | 0.91  (0.76-1.08) | 1.07  (0.85-1.35) | 1.11  (0.86-1.41) | 1.37  (1.05-1.78) |  |

The adjusted hazard ratio was calculated using Cox proportional hazards regression after adjusting age, sex, household income, smoking, physical activity, body mass index, systolic blood pressure, triglyceride, fasting serum glucose, total cholesterol and Charlson comorbidity index.
